# Social Isolation and Health Outcomes in Japan: A Nationwide Cross-Sectional Study

**DOI:** 10.31662/jmaj.2026-0129

**Published:** 2026-05-29

**Authors:** Hiroshi Ito, Shuichi Okaya, Tatsuya Watanabe, Ying-Fang Zheng, Nobutake Shimojo, Satoru Kawano, Takahiro Tabuchi

**Affiliations:** 1 Department of Hospital Medicine, University of Tsukuba Hospital, Tsukuba, Japan; 2 Health Services Research and Development Center, University of Tsukuba, Tsukuba, Japan; 3 Department of Emergency and Critical Care Medicine, University of Tsukuba Hospital, Tsukuba, Japan; 4 Department of Internal Medicine, Ibaraki Prefectural University of Health Sciences Hospital, Inashiki, Japan; 5 Division of Epidemiology, School of Public Health, Tohoku University Graduate School of Medicine, Sendai, Japan

**Keywords:** social isolation, symptom burden, chronic disease, healthcare-seeking behaviors, Lubben Social Network Scale-6

## Abstract

**Introduction:**

Social isolation is an important social determinant of health, but its relationship with health-related outcomes remains incompletely understood. This study examined whether structural social isolation is associated with subjective health, symptom burden, chronic disease diagnoses, and healthcare-seeking behaviors in a nationwide Japanese population.

**Methods:**

We conducted a nationwide cross-sectional study using data from the 2024 Japan coronavirus disease 2019 and Society Internet Survey, including adults aged 18-79 years. Social isolation was assessed using the Lubben Social Network Scale-6. Primary outcomes were poor self-rated health and high symptom burden, defined by the Somatic Symptom Scale-8. Secondary outcomes included self-reported chronic diseases and healthcare-seeking behaviors.

**Results:**

Among 25,014 participants (mean age: 50.0 years; 49.9% men), 59.3% were classified as socially isolated. Social isolation was associated with higher odds of poor self-rated health (adjusted odds ratio [AOR]: 1.54, 95% confidence interval [CI]: 1.32-1.81) and high symptom burden (AOR: 1.23, 95% CI: 1.09-1.38). In contrast, social isolation was not associated with a higher prevalence of most chronic diseases. Socially isolated individuals were also less likely to have a regular doctor or receive preventive services.

**Conclusions:**

Structural social isolation was associated with poorer subjective health and greater symptom burden but not with a higher prevalence of diagnosed chronic diseases. These findings suggest that reliance on diagnosis-based indicators alone may underestimate the health needs of socially isolated individuals, even in healthcare systems with broad accessibility.

## Introduction

Social isolation is a major social determinant of health, with a magnitude of risk comparable to that of traditional lifestyle-related factors such as smoking and physical inactivity ^(1), (2), (3)^. A growing body of evidence from cohort studies and meta-analyses has linked social isolation to increased all-cause mortality, cardiovascular disease, and adverse mental health outcomes, highlighting the broad health consequences of diminished social connections ^(1), (4), (5)^. Reflecting this evidence, the World Health Organization has identified social isolation and loneliness as emerging global public health concerns and has called for coordinated actions to strengthen social connections ^(6)^.

Despite this recognition, social isolation is a multidimensional construct that has been inconsistently operationalized across studies. It encompasses at least two related but distinct dimensions: subjective loneliness, reflecting perceived deficiencies in social connection, and structural social isolation, which captures objective aspects of social relationships such as social network size, contact frequency, and living arrangements ^(7), (8), (9)^. Measures of structural social isolation vary widely, ranging from validated social network scales, such as the Lubben Social Network Scale (LSNS) and the Berkman-Syme Social Network Index, to single indicators such as living alone, limiting comparability across studies ^(10)^. Moreover, prior research has largely focused on hard outcomes such as mortality and cardiovascular events, whereas associations with symptom burden and the broader prevalence of chronic diseases have received far less empirical attention ^(7), (11)^.

An additional concern is that socially isolated individuals may be less likely to engage in healthcare-seeking behaviors, even when experiencing health problems. Previous studies have shown associations between social isolation or loneliness and reduced use of healthcare services, as well as higher levels of unmet healthcare needs ^(12), (13), (14)^. As a result, socially isolated individuals may experience significant symptoms or declines in subjective health while being less likely to receive formal diagnoses of chronic diseases, suggesting that reliance on diagnosis-based outcomes may underestimate their true health burden. However, few studies have simultaneously examined symptom burden, chronic disease prevalence, and healthcare-seeking behaviors within the same population.

Japan provides a unique context to examine this potential mismatch, as its universal health coverage system with a free-access model allows patients to seek care at any medical institution without referral, resulting in some of the highest outpatient visit rates among Organisation for Economic Co-operation and Development (OECD) countries ^(15), (16)^. In this setting, system-level barriers to healthcare access are relatively low. In this study, we used data from a nationwide internet-based survey to assess the associations between structural social isolation, as measured by the LSNS-6, and a range of health-related outcomes―including self-reported health status, symptom burden, chronic disease prevalence, and healthcare-seeking behaviors―among adults in Japan.

## Materials and Methods

### Study design and setting

This cross-sectional study analyzed data from the Japan coronavirus disease 2019 (COVID-19) and Society Internet Survey (JACSIS), a nationwide, web-based, self-administered questionnaire conducted by Rakuten Insight, Inc., a major internet research company ^(17)^. As of the survey period, Rakuten Insight maintained a panel of approximately 2.2 million registered individuals across Japan. In collaboration with the company, we recruited participants using procedures designed to approximate the demographic distribution of the general population.

The 2024 JACSIS survey collected information on sociodemographic characteristics, lifestyle factors, health status, and healthcare utilization from 28,000 individuals aged 15-79 years. We excluded respondents aged 15-17 years, those who failed an attention-check question, those who reported use of all listed illicit substances, and those who selected all disease categories in the medical history section, as these responses were considered implausible (Supplementary Table S1). Because the analytic sample included newly recruited participants, a single overall response rate for the study population could not be defined.

The study protocol was reviewed and approved by the Research Ethics Committee of the Osaka International Cancer Institute (approval number 20084-2) prior to survey initiation and was subsequently updated and approved by the Ethics Committee of the University of Tsukuba Hospital (approval number R07-178). This study was conducted in accordance with the Declaration of Helsinki and is reported following the Strengthening the Reporting of Observational Studies in Epidemiology guidelines.

### Exposure variable: structural social isolation

The primary exposure was structural social isolation, assessed using the LSNS-6. The LSNS-6 is a validated six-item instrument designed to measure the size and frequency of social contacts with family members and friends. Originally developed to assess social isolation among older adults ^(18)^, the LSNS-6 has been translated and validated in Japanese populations ^(19)^ and has subsequently been applied in studies involving younger and middle-aged adults ^(20), (21), (22)^. Each item is scored on a six-point scale, yielding a total score ranging from 0 to 30, with lower scores indicating smaller and less frequent social networks (Supplementary Table S2). Consistent with prior studies, participants with an LSNS-6 score of less than 12 were classified as socially isolated, whereas those with a score of 12 or higher were classified as socially connected. In the main analyses, structural social isolation was treated as a binary exposure variable.

### Outcome variables

Primary outcomes were self-reported health status and symptom burden at the time of the survey. Self-reported health status was assessed using a standard single-item question. Respondents in the 2024 survey were asked, “How is your current health condition? Please choose only one answer that applies to you.” Poor health status was defined as selecting “Not very good” or “Not good.” Respondents who selected “Good,” “Fairly good,” or “Average” were classified as not having poor health status. Symptom burden was evaluated using the Somatic Symptom Scale-8 (SSS-8), a validated measure of somatic symptom severity that has also been validated in its Japanese version ^(23), (24)^. Respondents were asked, “Over the past week, how much have you been bothered by the following physical problems?” followed by eight symptoms: (1) stomach or bowel problems; (2) back pain; (3) pain in arms, legs, or joints; (4) headaches; (5) chest pain or shortness of breath; (6) dizziness; (7) feeling tired or having low energy; and (8) trouble sleeping. Each item was rated on a five-point scale ranging from 0 (“Not at all”) to 4 (“Very much”), yielding a total score ranging from 0 to 32. High symptom burden was defined as an SSS-8 score of 13 or higher, consistent with established cutoffs.

Secondary outcomes included physician-diagnosed chronic diseases and healthcare-seeking behaviors. Chronic diseases were assessed based on self-reported physician diagnoses and included hypertension, diabetes mellitus, dyslipidemia, coronary artery disease, stroke, cancer, chronic kidney disease, chronic obstructive pulmonary disease, asthma, atopic dermatitis, liver disease, and mental disorders. Having the corresponding disease was defined as selecting “Present now.” Respondents who selected “Not present” or “Not present now but in the past” were classified as not having the disease. Healthcare-seeking behaviors included having a regular source of care (family doctor), receipt of routine health checkups, receipt of dental checkups, and vaccination in the previous year. All healthcare-seeking behaviors were assessed using binary (yes/no) responses.

### Covariates

We adjusted for demographic, socioeconomic, and lifestyle-related variables that could potentially confound the association between structural social isolation and health outcomes. For each outcome, we constructed three prespecified weighted multivariable logistic regression models. Model 1 adjusted for demographic and socioeconomic factors. Model 2 additionally adjusted for lifestyle-related factors, and Model 3 further included healthcare-seeking behaviors, except when healthcare-seeking behaviors themselves were specified as outcome variables.

Demographic variables included age (categorized into 10-year intervals), sex, and residential region. Residential region was categorized as eastern Japan (Hokkaido, Tohoku, and Kanto), central Japan (Chubu and Kinki), or western Japan (Chugoku, Shikoku, Kyushu, and Okinawa). Socioeconomic factors included academic attainment (college or higher vs. high school or lower), equivalent household income, occupation class (upper nonmanual workers, lower nonmanual workers, manual workers, farmers, self-employed, and unemployed) ^(25)^, marital status (married, unmarried, widowed, or separated), and household size (1, 2, 3, 4, or ≥5 members). Equivalent household income was calculated by dividing total household income by the square root of household size and was categorized into quintiles from lowest (1st) to highest (5th), with an additional category for missing responses ^(26)^. These variables were selected from among socioeconomic indicators that have been widely used and summarized in previous research, focusing on those that were measurable within the JACSIS survey ^(27), (28), (29)^.

Lifestyle-related factors included body mass index (<18.5, 18.5-24.9, or ≥25.0 kg/m^2^), smoking status (never, ever, or current), alcohol consumption (never, <20 g/day, 20-40 g/day, or ≥40 g/day), daily walking time (<60 minutes/day), exercise duration (<30 minutes/day), and sleep duration (<6 hours/day), whose cutoffs were defined in accordance with recommendations from Japan’s national health promotion policy, Health Japan 21 ^(30)^.

Healthcare-seeking behaviors―including having a family doctor, receipt of routine health checkups, receipt of dental checkups, and vaccination in the previous year―were defined as described in the “Outcome variables” section.

### Statistical analysis

First, we summarized the characteristics of participants according to social isolation status. To address potential selection bias inherent in internet-based surveys, inverse probability weighting was applied using the 2019 Comprehensive Survey of Living Conditions as the reference population ^(31)^. The weighting procedure followed the approach used in previous JACSIS studies to align the demographic distribution of the survey sample with that of the general Japanese population ^(32)^. Details of the weighting methodology have been described previously ^(33)^. In addition, inverse probability-of-attrition weights were constructed using information on exclusion criteria, and all analyses were conducted using the combined weights.

We then examined associations between structural social isolation and each outcome using weighted multivariable logistic regression models. All models were adjusted for the covariates described above. Adjusted odds ratios (AORs) and 95% confidence intervals (CIs) were calculated, with socially connected participants serving as the reference group. A two-sided p-value of <0.05 was considered statistically significant. All analyses were conducted using Stata version 15 (StataCorp LLC, College Station, TX, USA).

### Sensitivity analyses

As a sensitivity analysis, we repeated all regression analyses without applying inverse probability weighting (unweighted models) to assess the robustness of the findings to the weighting procedure. In additional analyses, we examined potential dose-response relationships between structural social isolation and the primary outcomes using alternative categorizations of LSNS-6 scores. Specifically, associations between LSNS-6 five equal-width categories (0-5, 6-11, 12-17, 18-23, and ≥24) and the primary outcomes were estimated using inverse probability-weighted logistic regression models, adjusted according to Model 1 covariates, and the results were summarized using forest plots; the p-value for this coefficient was reported as p for trend.

### Patient and public involvement

Participants were not involved in the design, conduct, reporting, or dissemination plans of this research. Informed consent was obtained electronically from all participants prior to their participation.

## Results

### Participant characteristics

Of the 28,000 individuals who responded to the 2024 JACSIS survey, 25,014 participants aged 18-79 years were included in the analytic sample after excluding respondents aged 15-17 years and those with invalid responses (Supplementary Figure S1). The mean (standard deviation) age was 50.0 (30.4) years, and 49.9% were men (Table 1). Overall, 14,826 participants (59.3%) were classified as socially isolated based on LSNS-6 scores. Compared with socially connected participants, those with structural social isolation were less likely to have a college education, more likely to have lower household income, and more likely to be unmarried and live alone. They were also more likely to report unhealthy lifestyle behaviors, such as insufficient physical activity and shorter sleep duration.

**Table 1.** Characteristics of the Participants.

Characteristics	Participants with social isolation (n = 14,826)	Participants without social isolation (n = 10,188)
Male, n (%)	7,685 (51.8)	4,799 (47.1)
Age (years), mean (SD)	50.3 (28.8)	49.6 (32.5)
Region, n (%)		
East Japan	5,924 (40.0)	3,828 (37.6)
Central Japan	5,324 (35.9)	3,720 (36.5)
West Japan	3,578 (24.1)	2,641 (25.9)
Academic attainment, n (%)		
College or higher	6,292 (42.4)	4,800 (47.1)
High school or lower	8,534 (57.6)	5,388 (52.9)
Equivalent household income, n (%)		
1st quintile (<1.8 million JPY)	2,064 (13.9)	1,102 (10.8)
2nd quintile (1.8-3.0 million JPY)	3,092 (20.9)	2,020 (19.8)
3rd quintile (3.0-4.0 million JPY)	2,059 (13.9)	1,465 (14.4)
4th quintile (4.0-5.5 million JPY)	2,137 (14.4)	1,629 (16.0)
5th quintile (≥5.5 million JPY)	1,599 (10.8)	1,433 (14.1)
Unanswered	3,876 (26.1)	2,538 (24.9)
Occupation, n (%)		
Upper nonmanual workers	1,332 (9.0)	1,196 (11.7)
Lower nonmanual workers	5,093 (34.4)	3,537 (34.7)
Manual workers	2,324 (15.7)	1,497 (14.7)
Farmers	106 (0.7)	104 (1.0)
Self-employed	822 (5.5)	660 (6.5)
Unemployed	5,149 (34.7)	3,195 (31.4)
Marital status, n (%)		
Married	9,226 (62.2)	6,701 (65.8)
Never married	4,055 (27.4)	2,354 (23.1)
Widowed	713 (4.8)	668 (6.6)
Separated	832 (5.6)	465 (4.6)
Household size (members), n (%)		
1	2,775 (18.7)	1,520 (14.9)
2	5,259 (35.5)	3,216 (31.6)
3	3,464 (23.4)	2,386 (23.4)
4	2,329 (15.7)	2,102 (20.6)
≥5	998 (6.7)	964 (9.5)
Body mass index (kg/m^2^), n (%)		
<18.5	1,935 (13.1)	1,220 (12.0)
18.5-24.9	9,842 (66.4)	7,123 (69.9)
≥25.0	3,049 (29.6)	1,845 (18.1)
Smoking, n (%)		
Never	2,939 (19.8)	3,113 (30.6)
Ever	7,138 (48.1)	4,259 (41.8)
Current	4,749 (32.0)	2,816 (27.6)
Alcohol, n (%)		
Never	7,945 (53.6)	4,802 (47.1)
≤20 g/day	3,213 (21.7)	2,521 (24.7)
20-40 g/day	1,900 (12.8)	1,593 (15.6)
>40 g/day	1,768 (11.9)	1,271 (12.5)
Walking <60 minutes/day, n (%)	7,127 (48.1)	4,200 (41.2)
Exercising <30 minutes/day, n (%)	1,1961 (80.7)	7,126 (69.9)
Sleeping <6 hours/day, n (%)	4,062 (27.4)	2,277 (22.3)
Comorbidities, n (%)		
Hypertension	3,515 (23.7)	2,555 (25.1)
Periodontal diseases	2,492 (16.8)	1,630 (16.0)
Dyslipidemia	2,248 (15.2)	1,726 (16.9)
Dental caries	2,149 (14.5)	1,219 (12.0)
Diabetes mellitus	1,206 (8.1)	986 (9.7)
Mental diseases	1,237 (8.3)	571 (5.6)
Asthma	623 (4.2)	529 (5.2)
Coronary artery diseases	403 (2.7)	459 (4.5)
Malignancies	439 (3.0)	411 (4.0)
Stroke	299 (2.0)	258 (2.5)
COPD	253 (1.7)	241 (2.4)

Notes: We applied inverse probability weighting to account for selection into the internet survey and the exclusion of ineligible cases. COPD: chronic obstructive pulmonary disease; JPY: Japanese yen.

### Structural social isolation and subjective health outcomes

**Figure 1.**
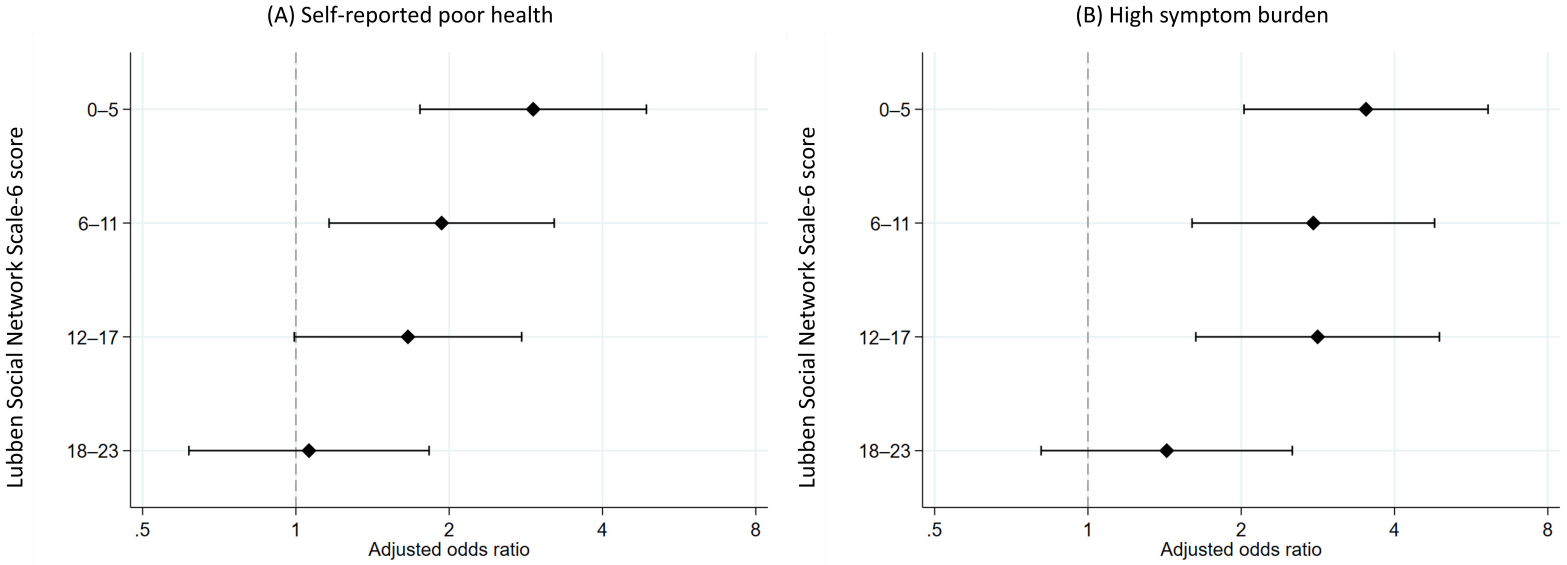
Association between LSNS-6 and subjective health status. The analyses applied inverse probability weighting to account for selection into the internet survey and the exclusion of ineligible cases. For each outcome, we constructed weighted multivariable logistic regression models adjusted for demographic and socioeconomic status. We categorized the LSNS-6 score into five groups (0-5, 6-11, 12-17, 18-23, and 24-30 [reference]) for visualization in contrast to the primary analyses using a dichotomized LSNS-6 measure (score <12 [socially isolated] vs. ≥12 [socially connected]). A significant dose-response relationship was observed for both self-reported poor health and high symptom burden (p for trend < 0.001 for both). LSNS-6: Lubben Social Network Scale-6.

After adjustment for demographic and socioeconomic factors, participants with structural social isolation were more likely to report poor health (AOR: 1.57, 95% CI: 1.39-1.78) and high symptom burden (AOR: 1.30, 95% CI: 1.16-1.45) than socially connected participants (Table 2). These associations remained robust after additional adjustment for lifestyle-related factors and healthcare-seeking behaviors (AOR for poor self-reported health: 1.54, 95% CI: 1.32-1.81; AOR for high symptom burden: 1.23, 95% CI: 1.09-1.38). When LSNS-6 scores were categorized into five equal-width groups, a graded dose-response association was observed, with lower LSNS-6 scores (indicating greater social isolation) associated with higher odds of poor self-reported health and high symptom burden (Figure 1).

**Table 2.** Association between Social Isolation and Subjective Health Status.

Variables	Participants with social isolation (n = 14,826), n (%)	Participants without social isolation (n = 10,188), n (%)	Model 1			Model 2			Model 3		
			AOR	95% CI	p-Value	AOR	95% CI	p-Value	AOR	95% CI	p-Value
Self-reported poor health	2,250 (15.2)	1,023 (10.0)	1.57	1.39-1.78	<0.001	1.49	1.28-1.75	<0.001	1.54	1.32-1.81	<0.001
High symptom burden*	3,076 (20.7)	1,764 (17.3)	1.30	1.16-1.45	<0.001	1.24	1.10-1.40	<0.001	1.23	1.09-1.38	0.001

Notes: The analyses applied inverse probability weighting to account for selection into the internet survey and the exclusion of ineligible cases. For each outcome, we constructed three prespecified weighted multivariable logistic regression models. Model 1 adjusted for demographic and socioeconomic factors. Model 2 additionally adjusted for health-related status, and Model 3 further included healthcare-seeking behaviors. *High symptom burden was defined as Somatic Symptom Scale 8 score ≥13 points. AOR: adjusted odds ratio; CI: confidence interval.

### Structural social isolation and chronic diseases

Structural social isolation was not associated with a higher prevalence of most self-reported chronic diseases (Table 3). In fully adjusted models, social isolation was inversely associated with several chronic conditions, including coronary artery disease (AOR: 0.63, 95% CI: 0.48-0.83) and diabetes mellitus (AOR: 0.84, 95% CI: 0.71-0.99). In contrast, social isolation was positively associated with mental disorders (AOR: 1.51, 95% CI: 1.26-1.79).

**Table 3.** Association between Social Isolation and Self-Reported Comorbidities.

Variables	Participants with social isolation (n = 14,826), n (%)	Participants without social isolation (n = 10,188), n (%)	Model 1			Model 2			Model 3		
			AOR	95% CI	p-Value	AOR	95% CI	p-Value	AOR	95% CI	p-Value
Hypertension	3,515 (23.7)	2,555 (25.1)	0.91	0.82-1.02	0.10	0.90	0.80-1.01	0.07	0.97	0.87-1.09	0.66
Periodontal diseases	2,492 (16.8)	1,630 (16.0)	1.02	0.90-1.15	0.78	0.99	0.87-1.11	0.83	1.02	0.90-1.15	0.79
Dyslipidemia	2,248 (15.2)	1,726 (16.9)	0.85	0.76-0.96	0.008	0.83	0.74-0.94	0.003	0.89	0.79-1.01	0.06
Dental caries	2,149 (14.5)	1,219 (12.0)	1.16	1.03-1.32	0.02	1.11	0.98-1.25	0.11	1.07	0.94-1.22	0.29
Diabetes mellitus	1,206 (8.1)	986 (9.7)	0.82	0.70-0.96	0.02	0.80	0.67-0.94	0.007	0.84	0.71-0.99	0.04
Mental diseases	1,237 (8.3)	571 (5.6)	1.43	1.21-1.69	<0.001	1.37	1.15-1.63	<0.001	1.51	1.26-1.79	<0.001
Asthma	623 (4.2)	529 (5.2)	0.82	0.68-0.99	0.04	0.82	0.68-1.00	0.05	0.87	0.71-1.06	0.17
Coronary artery diseases	403 (2.7)	459 (4.5)	0.60	0.47-0.77	<0.001	0.62	0.47-0.81	0.001	0.63	0.48-0.83	0.001
Malignancies	439 (3.0)	411 (4.0)	0.79	0.62-1.00	0.052	0.79	0.61-1.02	0.07	0.80	0.61-1.04	0.09
Stroke	299 (2.0)	258 (2.5)	0.84	0.62-1.14	0.27	0.93	0.68-1.27	0.65	0.95	0.69-1.30	0.74
COPD	253 (1.7)	241 (2.4)	0.76	0.57-1.02	0.07	0.86	0.63-1.18	0.33	0.86	0.63-1.17	0.33

Notes: The analyses applied inverse probability weighting to account for selection into the internet survey and the exclusion of ineligible cases. For each outcome, we constructed three prespecified weighted multivariable logistic regression models. Model 1 adjusted for demographic and socioeconomic factors. Model 2 additionally adjusted for health-related status, and Model 3 further included healthcare-seeking behaviors. AOR: adjusted odds ratio; CI: confidence interval; COPD, chronic obstructive pulmonary disease.

### Structural social isolation and healthcare-seeking behaviors

Participants with structural social isolation were less likely to engage in healthcare-seeking behaviors (Table 4). Specifically, social isolation was associated with lower odds of having a family doctor (AOR: 0.65, 95% CI: 0.60-0.72), receiving annual health checkups (AOR: 0.80, 95% CI: 0.73-0.89), receiving annual dental checkups (AOR: 0.77, 95% CI: 0.71-0.85), and receiving influenza or COVID-19 vaccination in the previous year (AOR: 0.88, 95% CI: 0.81-0.97).

**Table 4.** Association between Social Isolation and Healthcare-Seeking Behaviors.

Variables	Participants with social isolation (n = 14,826), n (%)	Participants without social isolation (n = 10,188), n (%)	Model 1			Model 2		
			AOR	95% CI	p-Value	AOR	95% CI	p-Value
Having a family doctor	6,559 (44.2)	5,618 (55.1)	0.65	0.59-0.71	<0.001	0.65	0.60-0.72	<0.001
Receiving annual health checkups	10,258 (69.2)	7,567 (74.3)	0.79	0.72-0.87	<0.001	0.80	0.73-0.89	<0.001
Receiving annual dental checkups	8,051 (54.3)	6,377 (62.6)	0.75	0.68-0.81	<0.001	0.77	0.71-0.85	<0.001
Influenza or COVID-19 vaccination in the previous year	6,541 (44.1)	5,003 (49.1)	0.86	0.79-0.94	0.001	0.88	0.81-0.97	0.008

Notes: The analyses applied inverse probability weighting to account for selection into the internet survey and the exclusion of ineligible cases. For each outcome, we constructed three prespecified weighted multivariable logistic regression models. Model 1 adjusted for demographic and socioeconomic factors. Model 2 additionally adjusted for health-related status. AOR: adjusted odds ratio; CI: confidence interval.

### Sensitivity analyses

The main findings were qualitatively unchanged in analyses conducted without inverse probability weighting (Supplementary Tables S3-S5).

## Discussion

In this nationwide cross-sectional study of adults in Japan, we found that structural social isolation was consistently associated with poorer subjective health and greater symptom burden, while it was not associated with a higher prevalence of most physician-diagnosed chronic diseases and was inversely associated with several conditions. In contrast, socially isolated individuals were markedly less likely to engage in healthcare-seeking behaviors, including having a regular source of care, receiving routine health checkups, and obtaining preventive services. Notably, these patterns were observed in Japan, a country characterized by high healthcare accessibility and a free-access medical system. Taken together, our findings indicate a potential mismatch between experienced health problems and diagnosed chronic conditions among socially isolated individuals, suggesting that health needs in this population may not be fully captured by diagnosis-based measures, although these findings should be interpreted with caution given the reliance on self-reported diagnoses.

Several mechanisms may account for the observed pattern whereby structural social isolation was associated with greater symptom burden and poorer subjective health but not with a higher prevalence of physician-diagnosed chronic diseases. Socially isolated individuals may have fewer opportunities or resources to engage with healthcare systems, reflecting reduced social support and enabling factors for accessing care ^(34)^. Prior studies have shown that social isolation is associated with unmet medical needs and a greater reliance on alternative forms of medical care, even in the presence of health problems ^(14), (35)^. As a result, socially isolated individuals may experience persistent symptoms or functional impairments while remaining less likely to receive formal diagnoses.

Furthermore, social isolation has been linked to adverse health through behavioral and psychosocial pathways, including reduced physical activity, poor sleep, and heightened psychological stress, which may exacerbate symptom burden and worsen perceived health without necessarily leading to clinical diagnoses ^(5), (36)^. In contrast, conditions that are accompanied by salient or distressing symptoms―such as mental disorders or dental problems―may be more readily perceived by individuals themselves and thus more likely to be identified even in the absence of regular healthcare engagement. However, the association between structural social isolation and diagnosed chronic diseases is likely bidirectional and dynamic. Individuals with a higher burden of diagnosed chronic diseases may have more frequent contact with healthcare professionals, family members, or other support networks because of their illness, potentially resulting in larger observed social networks despite poorer underlying health ^(37)^. Consistent with this interpretation, adjustment for healthcare-seeking behaviors in our analysis modestly attenuated the inverse associations observed for some chronic diseases, suggesting that reduced engagement with healthcare may partially mediate or confound the relationship between social isolation and diagnosed conditions. Therefore, the observed lack of positive association between social isolation and diagnosed chronic diseases may reflect differences in healthcare engagement rather than true differences in underlying disease prevalence.

Japan provides a distinctive context for examining the relationship between social isolation and health. Under a universal health coverage system with a free-access model, individuals can generally seek care at medical institutions of their choice, and outpatient utilization in Japan is among the highest in OECD countries ^(38), (39)^. This suggests that institutional barriers to accessing outpatient care are relatively low in international comparison. Against this backdrop, our finding that structural social isolation was associated with poorer subjective health and greater symptom burden yet showed limited or inverse associations with physician-diagnosed chronic diseases and healthcare-seeking behaviors is noteworthy. These results indicate that even in a healthcare system characterized by broad coverage and high accessibility, social isolation is associated with a disconnect between experienced health problems and medical encounters or formal diagnoses. Thus, high system-level accessibility alone may not be associated with effective connection of all individuals with health needs to healthcare services. In countries with stricter gatekeeping, higher out-of-pocket costs, or longer waiting times, the disconnect between symptom burden and diagnosed disease among socially isolated individuals may be even more pronounced ^(39)^. Nevertheless, as this study was conducted within a specific institutional and cultural context, caution is warranted in generalizing these findings internationally, underscoring the need for comparative studies across healthcare systems with differing access structures.

Our findings indicate that structural social isolation should be viewed not only as a social concern but also as a challenge to the recognition of health problems. Approaches that rely primarily on diagnosis-based indicators or healthcare utilization may underestimate the true burden of symptoms and poor perceived health among socially isolated individuals, even in healthcare systems with broad coverage. From a clinical perspective, these results suggest that the incorporation of social isolation screening into routine primary care and preventive health services may be warranted. Expert groups have increasingly emphasized the role of healthcare settings in identifying social isolation as a risk factor for adverse health outcomes ^(40)^. Beyond screening, our findings also highlight the need for closer integration between healthcare services and community-based social resources. Prior research suggests that interventions addressing social isolation are more effective when primary care is linked with public health and nonmedical community organizations, including social prescribing and group-based engagement programs ^(41), (42)^. Such strategies may facilitate earlier engagement with care and help mitigate symptom burden, even when formal diagnoses have not yet been established.

Several limitations should be acknowledged. First, regarding generalizability, this study was based on a nationwide, internet-based survey conducted in Japan, and the findings may not be directly applicable to populations in countries with different healthcare systems or social contexts. Although inverse probability weighting was used to enhance representativeness, individuals who are socially isolated and entirely disconnected from online environments may remain underrepresented. Second, potential sources of internal bias should be considered. Selection bias may persist due to the voluntary nature of participation, and reporting bias is possible because both social isolation and health outcomes were self-reported. Chronic diseases were defined based on self-reported physician diagnoses, which may reflect healthcare utilization as much as underlying disease prevalence, and thus should be interpreted with caution. Residual confounding cannot be excluded despite adjustment for a broad set of demographic, socioeconomic, lifestyle-related, and healthcare-seeking factors. Additionally, residential region was categorized using broad geographic groupings rather than more detailed contextual indicators such as population density or healthcare resource availability, which may limit the ability to fully account for regional variation. Third, the cross-sectional design precludes causal inference. The observed null or inverse associations between structural social isolation and several diagnosed chronic diseases cannot be fully explained within this study. While reduced engagement with healthcare among socially isolated individuals may contribute to underrecognition or underdiagnosis, reverse causation―whereby individuals with diagnosed chronic diseases have increased social contact through healthcare or support networks―also remains plausible. Longitudinal studies are needed to clarify the temporal and causal relationships underlying these associations. Finally, because the data were derived from the 2024 JACSIS survey, social behaviors and healthcare-seeking patterns may have been influenced by the broader context of the COVID-19 pandemic, and future studies are needed to confirm whether these findings persist in nonpandemic settings.

## Conclusions

In this nationwide cross-sectional study, structural social isolation was consistently associated with poorer subjective health and greater symptom burden but not with a higher prevalence of most physician-diagnosed chronic diseases, even in a healthcare system characterized by universal coverage and high accessibility. Socially isolated individuals were also less likely to engage in healthcare-seeking behaviors, suggesting that reliance on diagnosis-based indicators alone may underestimate the true health burden in this population. These findings should be interpreted with caution, as self-reported diagnoses may reflect differences in healthcare engagement rather than true differences in disease prevalence. Taken together, the results highlight social isolation as an important determinant not only of health outcomes but also of health problem recognition. Integrating the assessment of social isolation into primary care and preventive health services, alongside stronger linkages between healthcare and community-based resources, may help improve the detection and management of unmet health needs among socially isolated individuals.

## Article Information

### Acknowledgments

The data used in this study were originally collected under the project (the 2024 Japan COVID-19 and Society Internet Survey) supported by the Japan Society for the Promotion of Science KAKENHI Grants (grant numbers JP21H04856; JP25H01079 [Takahiro Tabuchi]; and JP24K15576 [Kanami Tsuno]), Japan Science and Technology Agency Research Institute of Science and Technology for Society grants (grant number JPMJRX21K6 [Midori Matsushima]), the intramural fund of the National Institute for Environmental Studies (Shoji Nakayama), the Health Labor Sciences Research Grants (grant numbers 23EA1003 [Masayoshi Zaitsu]; 22FA2001 [Daisuke Nishi]; 24LA0201 [Atsushi Nakagomi]; 22JA1005; 22FA1001; 22FA1010; 23EA1001; 23FA1004; 24FA1020; and 24LA1002 [Takahiro Tabuchi]), and the Japan Agency for Medical Research and Development “Strategic Center of Biomedical Advanced Vaccine Research and Development for Preparedness and Response” (grant number JP243fa627004 [Yuki Furuse]).

### Author Contributions

Hiroshi Ito: conceptualization, methodology, data curation, formal analysis, investigation, writing - original draft, writing - review and editing, funding acquisition, and project administration. Shuichi Okaya, Tatsuya Watanabe, Ying-Fang Zheng, Nobutake Shimojo, and Satoru Kawano: methodology, investigation, and writing - review and editing. Takahiro Tabuchi: resources, supervision, and writing - review and editing. All authors approved the final manuscript and agreed to be accountable for all aspects of the work.

### Conflicts of Interest

Takahiro Tabuchi received financial support for research (research funding, consulting fees, or lecture fees) from Daiichi Sankyo Healthcare Co., Ltd., Johnson & Johnson K.K., Data Seed Inc., Workout-Plus LLC, and EMMA Co., Ltd. The other authors have no conflicts of interest to declare.

### Ethics Approval

The study protocol was reviewed and approved by the Research Ethics Committee of the Osaka International Cancer Institute (approval number 20084-2) prior to survey initiation and was subsequently updated and approved by the Ethics Committee of the University of Tsukuba Hospital (approval number R07-178).

## Supplement

Supplementary Material
