# Supplementary material for: Social Isolation and Health Outcomes in Japan: A Nationwide Cross-Sectional Study
Source: JMA J. 2026 May 29;9(4):815–24. doi: 10.31662/jmaj.2026-0129 (PMC13453561; doi:10.31662/jmaj.2026-0129)

Supplementary Material

Supplementary Table S1: Definition of invalid responses in the 2024 JACSIS survey

**Inaccurate responses include the following three definitions:**
(1) those who selected “yes” to all disease lists (do you have the following diseases? [more than ten items listed below]: hypertension, diabetes, asthma, bronchitis and pneumonia, atopic dermatitis, periodontal disease, caries, otitis media, coronary artery disease, stroke, chronic obstructive pulmonary disease), cancer, chronic pain such as back pain and headaches, depression, and psychiatric disorders other than depression
and/or
(2) those who selected “yes” to all drug use (do you use the following drugs?: alcohol, sleeping pills and/or anti-anxiety medications, morphine (three types), thinner, marijuana, dangerous drugs (e.g., outlawed herbs, magic mushrooms, etc.) and cocaine and/or heroin (nine items))
and/or
(3) those who selected other than “D” out of “A B C D E” in the question of “please select the second option from the bottom.”

Supplementary Table S2: The Lubben Social Network Scale-6

Items	Categories (score)					
	0	1	2	3	4	5
**Family: Considering the people to whom you are related by birth or marriage**						
How many relatives do you see or hear from at least once a month?	Zero	One	Two	Three or four	Five to eight	Nine or more
How many relatives do you feel close to such that you could call on them for help?	Zero	One	Two	Three or four	Five to eight	Nine or more
How many relatives do you feel at ease with that you can talk about private matters?	Zero	One	Two	Three or four	Five to eight	Nine or more
**Friendship: Considering all or your friends, including those who live in your neighborhood**						
How many of your friends do you see or hear from at least once a month?	Zero	One	Two	Three or four	Five to eight	Nine or more
How many friends do you feel close to such that you could call on them for help?	Zero	One	Two	Three or four	Five to eight	Nine or more
How many friends do you feel at ease with that you can talk about private matters?	Zero	One	Two	Three or four	Five to eight	Nine or more

Supplementary Table S3: Association between social isolation and subjective health status (unweighted version)

	Participants with social isolation, n (%)	Participants without social isolation, n (%)	Model 1			Model 2			Model 3		
			AOR	95% CI	p value	AOR	95% CI	p value	AOR	95% CI	p value
Self-reported poor health	3114 (21.0)	1184 (11.6)	1.78	1.65-1.92	<0.001	1.74	1.56-1.94	<0.001	1.79	1.61-2.00	<0.001
High symptom burden	3275 (22.1)	1728 (17.0)	1.34	1.25-1.43	<0.001	1.25	1.16-1.35	<0.001	1.24	1.15-1.34	<0.001

In this sensitivity analysis, we used unweighted regression instead of weighted regression; otherwise, see the caption of Table 2.

Supplementary Table S4: Association between social isolation and self-reported comorbidities (unweighted version)

	Participants with social isolation, n (%)	Participants without social isolation, n (%)	Model 1			Model 2			Model 3		
			AOR	95% CI	p value	AOR	95% CI	p value	AOR	95% CI	p value
Hypertension	3275 (22.1)	2418 (23.8)	0.93	0.87-0.99	0.04	0.91	0.85-0.98	0.02	0.97	0.90-1.04	0.42
Periodontal diseases	2379 (16.0)	1505 (14.8)	1.06	0.99-1.15	0.10	1.03	0.96-1.11	0.43	1.05	0.97-1.13	0.25
Dyslipidemia	2298 (15.5)	1711 (16.8)	0.92	0.85-0.98	0.02	0.89	0.83-0.96	0.002	0.94	0.87-1.01	0.11
Dental caries	2010 (13.5)	1197 (11.8)	1.08	1.00-1.17	0.049	1.03	0.95-1.12	0.47	1.00	0.92-1.08	0.98
Diabetes mellitus	1148 (7.7)	868 (8.5)	0.91	0.82-0.99	0.046	0.87	0.79-0.96	0.008	0.90	0.82-1.00	0.052
Mental diseases	1365 (9.2)	616 (6.1)	1.32	1.19-1.46	<0.001	1.23	1.10-1.37	<0.001	1.34	1.20-1.49	<0.001
Asthma	643 (4.3)	527 (5.2)	0.83	0.73-0.93	0.002	0.83	0.73-0.94	0.003	0.85	0.75-0.96	0.01
Coronary artery diseases	393 (2.6)	399 (3.9)	0.66	0.57-0.77	<0.001	0.68	0.58-0.79	<0.001	0.68	0.59-0.80	<0.001
Malignancies	443 (3.0)	413 (4.1)	0.77	0.67-0.89	<0.001	0.77	0.66-0.89	<0.001	0.77	0.66-0.89	<0.001
Stroke	275 (1.9)	287 (2.8)	0.63	0.53-0.75	<0.001	0.69	0.57-0.83	<0.001	0.68	0.57-0.82	<0.001
COPD	261 (1.8)	264 (2.6)	0.68	0.57-0.81	<0.001	0.76	0.63-0.91	0.004	0.74	0.61-0.90	0.002

In this sensitivity analysis, we used unweighted regression instead of weighted regression; otherwise, see the caption of Table 3.

Supplementary Table S5: Association between social isolation and healthcare seeking behaviors (unweighted version)

	Participants with social isolation, n (%)	Participants without social isolation, n (%)	Model 1			Model 2		
			AOR	95% CI	p value	AOR	95% CI	p value
Having a family doctor	6574 (44.3)	5633 (55.4)	0.68	0.65-0.72	<0.001	0.68	0.64-0.72	<0.001
Receiving annual health checkups	10549 (71.1)	7782 (76.5)	0.82	0.77-0.87	<0.001	0.84	0.79-0.90	<0.001
Receiving annual dental checkups	8437 (56.9)	6668 (65.5)	0.75	0.71-0.79	<0.001	0.78	0.74-0.83	<0.001
Influenza or COVID-19 vaccination in the previous year	6783 (45.7)	5281 (51.9)	0.89	0.84-0.94	<0.001	0.91	0.86-0.96	0.001

In this sensitivity analysis, we used unweighted regression instead of weighted regression; otherwise, see the caption of Table 4.

Supplementary Figure S1: Selection of the participants

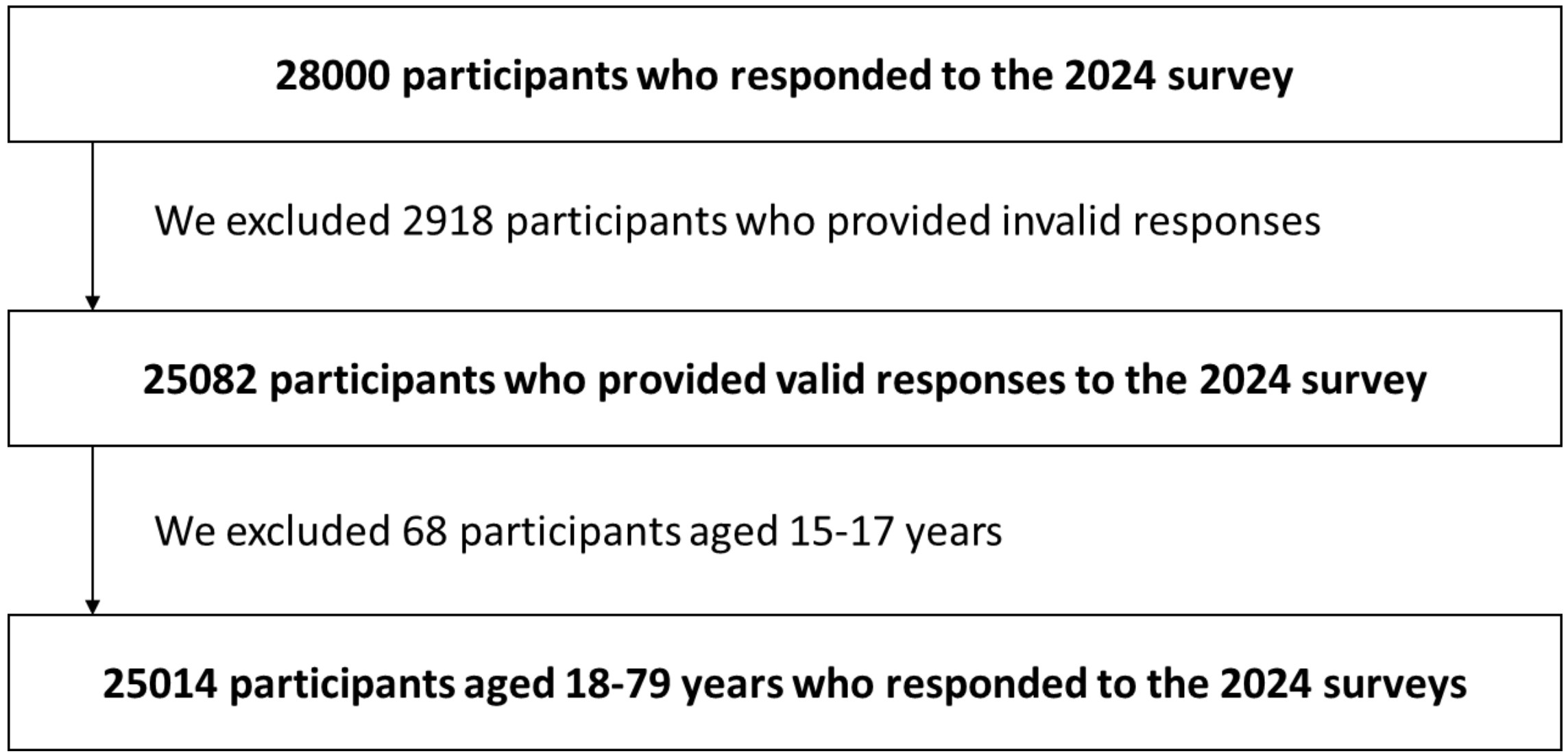

Supplement: Supplementary file 1 [file 2433-3298-9-4-0815-s001.pdf]
